# Design and synthesis of gold nanostars-based SERS nanotags for bioimaging applications

**DOI:** 10.7150/ntno.61244

**Published:** 2022-01-01

**Authors:** Bohdan Andreiuk, Fay Nicolson, Louise M. Clark, Sajanlal R. Panikkanvalappil, Kenry, Mohammad Rashidian, Stefan Harmsen, Moritz F. Kircher

**Affiliations:** 1 Department of Imaging, Dana-Farber Cancer Institute and Harvard Medical School, Boston, MA 02215, USA.; 2 Department of Cancer Immunology and Virology, Dana-Farber Cancer Institute and Harvard Medical School, Boston, MA 02215, USA.; 3 Department of Cancer Biology, Dana-Farber Cancer Institute and Harvard Medical School, Boston, MA 02215, USA.; 4 Harvard John A. Paulson School of Engineering and Applied Sciences, Harvard University, Cambridge, MA 02138, USA.; 5 Department of Radiology, Perelman School of Medicine, University of Pennsylvania, Philadelphia, PA 19104, USA.; 6 Department of Radiology, Brigham & Women's Hospital and Harvard Medical School, Boston, MA 022115, USA.

**Keywords:** Surface-enhanced Raman scattering, gold nanostar synthesis, near-infrared dye, cancer imaging

## Abstract

Surface-enhanced Raman spectroscopy (SERS) nanotags hold a unique place among bioimaging contrast agents due to their fingerprint-like spectra, which provide one of the highest degrees of detection specificity. However, in order to achieve a sufficiently high signal intensity, targeting capabilities, and biocompatibility, all components of nanotags must be rationally designed and tailored to a specific application. Design parameters include fine-tuning the properties of the plasmonic core as well as optimizing the choice of Raman reporter molecule, surface coating, and targeting moieties for the intended application. This review introduces readers to the principles of SERS nanotag design and discusses both established and emerging protocols of their synthesis, with a specific focus on the construction of SERS nanotags in the context of bioimaging and theranostics.

## Introduction

First reported in 1928, Raman spectroscopy is the phenomenon of inelastic scattering of light at longer or shorter wavelengths than that of the incident source 1, 2. Because such changes in frequency are specific to a particular vibration within the molecule of interest, the composition of the sample can be determined in a non-destructive manner using Raman spectroscopy 3. However, since only one in ~10^7^ photons scatters inelastically, Raman spectroscopy is an inherently weak technology. Interestingly, the Raman cross-section of a molecule can be significantly increased by adsorbing the molecule on a noble metal nanoparticle surface in a process known as surface-enhanced Raman scattering (SERS), which provides a means of overcoming the inherently weak nature of spontaneous Raman scattering.

SERS was first reported in 1974 by Fleischmann *et al.*, who observed an enhancement in the Raman scattering of pyridine adsorbed onto a chemically roughened silver electrode 4. SERS enhancement is attributed to two factors: electromagnetic enhancement and charge transfer. The former mechanism is dependent on an interaction between the localized surface plasmon resonance (LSPR) of the metallic nanoparticle surface and the adsorbed molecule 3. When incident laser light interacts with the metallic nanoparticles (NPs), it induces an oscillation of conduction band electrons, which increases polarizability of the adsorbed molecule leading to significant enhancement of Raman scattering 5. Enhancement factors of 10^7^-10^9^ have been reported 6.

**Figure 1:**
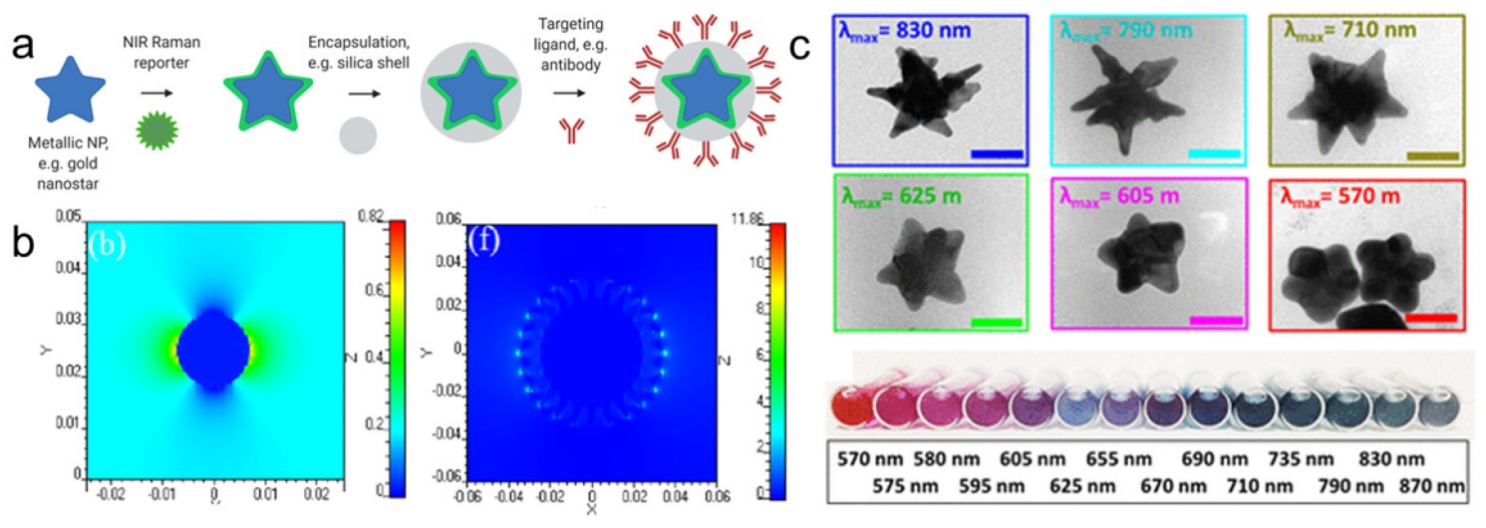
** Schematic representation of SERRS nanotag structure for *in vivo* imaging applications.** (a) Metallic substrates, e.g. gold nanostars, are functionalized with a NIR Raman reporter which are then encapsulated in a biocompatible coating such as silica. Active targeting can be achieved through conjugation of specific targeting moieties, e.g. antibodies to the nanoparticle surface. (b) 3D finite-difference time-domain (FDTD) simulated electromagnetic field distributions of a gold nanosphere (left) and a gold nanostar (right) excited with a 785 nm laser. Adapted with permission from ref. 16. (c) TEM images (top) and color in solution (bottom) of gold nanostars with different LSPR maxima. Adapted with permission from ref. 17.

Even greater enhancement in Raman signal can be achieved by placing molecules in so-called “hot spots” between NPs, where the electromagnetic field is maximized due to interaction of the localized surface plasmon resonances of neighboring NPs. Additionally, by adsorbing a molecule that has an absorption maximum that is resonant with the incident laser wavelength, additional enhancement is achieved in a process known as “surface-enhanced resonance Raman scattering” (SERRS) 7, 8. The observed SE(R)RS signal amplification is dependent on a number of factors including the interaction between adjacent NPs and their LSPRs, the number of analyte molecules on the surface of the metallic nanoparticle, and resonance contribution of both the analyte and metal. The resonance contribution of the metal is governed by the type, size, and shape of metallic nanoparticles 9, 10. For biomedical applications, SERS nanoparticles (or SERS nanotags) typically consist of a plasmonic nanoparticle core coated with molecules (Raman reporters) that are encapsulated in a protective shell that can be decorated with targeting ligands (Figure 1a). Raman reporters are selected not only based on their polarizability and resonance properties but also their binding affinity to the metal NP surface, which increases the overall number of reporters per particle, therefore generating higher signal intensity 3, 11, 12. The metal/molecule system is encapsulated in a biocompatible coating that stabilizes the metal nanoparticle and prevents desorption of the Raman reporter molecules from the NP's surface 13. Finally, the resulting SE(R)RS nanotags can be functionalized with targeting ligands that direct the nanoparticles towards specific disease markers to enable their detection using Raman imaging 12, 13.

While many have discussed the general principles and applications of SERS, here we will specifically discuss the important aspects of design and synthesis of SE(R)RS nanotags for biomedical applications, focusing on those with star-shaped plasmonic cores - nanostars 10, 11, 14, 15.

## Synthesis Strategies for Gold Nanostars

For biomedical applications, gold and silver nanoparticles are preferred, because their LSPRs are within the visible and near-infrared (NIR) regions of the light spectrum 11, 18. Although silver NPs exhibit stronger enhancement, their LSPR is blue-shifted relative to gold nanoparticles (AuNPs). Furthermore, due to their higher rates of biodegradation and release of toxic Ag^+^ ions, silver NPs are associated with poorer biocompatibility 19, 20. Since AuNPs are inert, biocompatible and their LSPRs can be shifted towards the NIR window - an optical window (700-950 nm) where tissue absorption and autofluorescence are minimized - AuNPs are regarded ideal for *in vivo* imaging applications 11. When carrying out *in vivo* imaging, a laser wavelength in the NIR region (e.g. 785 or 830 nm) is typically selected to ensure maximum tissue penetration 21. Through changes in their size and shape, the LSPR of the AuNPs can be tuned to be in resonance with the excitation laser wavelength 17. While spherical cores with a diameter of 10-100 nm have an LSPR of 530-580 nm, respectively, the LSPR of anisotropic AuNPs (*e.g.* rods, cubes, cages, or stars) are red-shifted into the NIR 13, 22-24. Among these core shapes, gold nanostars are known to generate the highest Raman enhancement factors (Figure 1b) 16, 25.

Many different protocols of nanostars synthesis have been reported 26-28. The outcomes and reproducibility of these synthesis protocols are affected by many factors, including, but not limited to the reducing, capping, and shape-directing agents used (with one reagent sometimes fulfilling multiple roles); the relative and absolute concentration as well as the oxidation state and ligands of the gold complexes used; the presence or absence of preformed seeds; reaction parameters like temperature, solvent and pH of the solution, scale of synthesis and mixing speed, and even the degree of glassware purity. Although it is highly desirable to obtain monodisperse nanoparticles during synthesis, it is possible to separate different shapes and sizes of nanoparticles by density gradient centrifugation 29 or field-flow fractionation. Below we will address the established gold nanostars synthesis methods and discuss their advantages and disadvantages with regards to their use in SERS-based bioimaging applications.

### Ascorbate-based synthesis

Ascorbic acid is one of the most commonly used reducing agents in nanostars synthesis and was first used to produce nanostars in 2004 30. This initial protocol required cetyltrimethylammonium bromide (CTAB), a cationic, shape-directing surfactant that is highly cytotoxic 31. Variations to this synthesis protocol have been reported where CTAB was replaced with sodium dodecyl sulfate (SDS) 32, albumin 33, benzylhexadecyldimethylammonium chloride (BDAC) and dioctyl sodium sulfosuccinate (AOT) 34, poly(diallyldimethylammonium chloride) (PDDA) 35, and nonionic surfactant Triton X-100 36.

**Figure 2:**
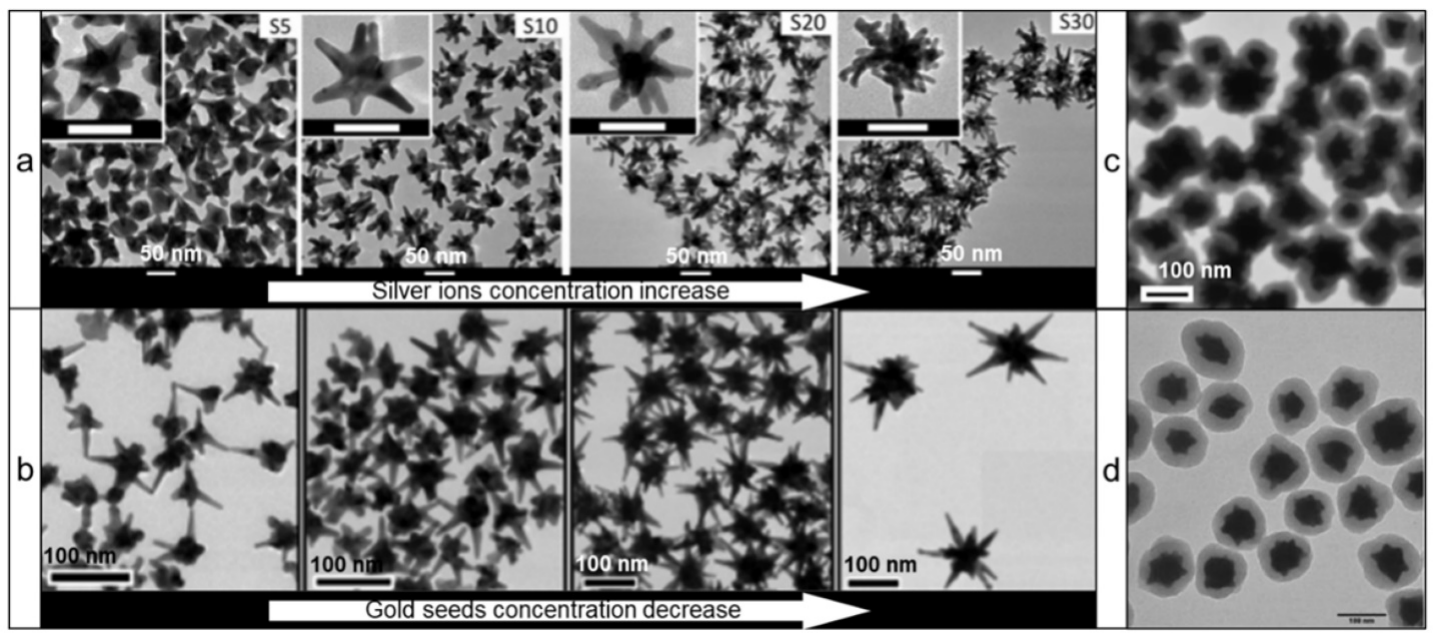
** Transmission electron microscopy (TEM) images of various gold nanostars synthesized via surfactant-free protocols using ascorbic acid as a reducing agent.** (a) Effect of silver concentration on the nanostar shape; adapted with permission from ref.^37^ (b) Effect of gold seeds concentration on the nanostar shape; adapted with permission from ref.^40^ Nanostars synthesized via (c) silver-free seedless and (d) seed-mediated protocols; adapted with permission from refs.^13^, ^42^

However, ascorbic acid's most important feature as a reducing agent is its ability to produce nanostars without any auxiliary surfactants. This is of great importance as stabilization of nanostars by auxiliary surfactants is achieved through adsorption to the NPs surface, which decreases the available surface area for Raman reporter adsorption and thus leads to diminished Raman signal intensity. In 2012, Vo-Dinh and co-workers described a surfactant-free gold nanostar synthesis protocol involving only gold seeds, gold ions, ascorbic acid, and small amounts of silver ions 37. Silver ions functioned as a shape-directing agent and varying quantities resulted in the preparation of stars of different shapes with red-shifted LSPRs (Figure 2a). This protocol has since been modified in numerous other reports (Figure 2b) 38-40.

More recently, Kircher and co-workers presented a seedless and silver-free protocol, which produced gold nanostars through rapid addition of gold ions to a solution of ascorbic acid at reduced temperatures (Figure 2c) 13, 26, 41. Since this method required rapid addition of gold ions to an ascorbic acid solution, it was difficult to scale-up; in larger volumes rapid homogeneous distribution of the gold ions across the entire volume was more difficult to achieve, leading to polydisperse nanostar preparations. This limitation was mitigated by using a seed-mediated version of ascorbate-based synthesis of gold nanostars (Figure 2d) 42.

### Silver as a shape-directing agent

Silver ions (Ag^+^) are a commonly used ingredient and act as a shape-directing agent in the synthesis of branched plasmonic nanomaterials, including gold nanostars. As in the case of gold nanorod crystal growth, it is likely that Ag^+^ ions can direct the anisotropic growth of gold nanoparticle seeds to form gold nanostars by preferential adsorption of silver halides onto the crystal facets of seeds, which restrict the growth of these passivated facets 43, 44. Yuan *et al.* demonstrated that the growth kinetics of seed nanoparticles can be tailored during seed-mediated growth approaches by incrementally varying the Ag^+^ concentration resulting in the formation of thorny three-dimensional (3D) nanoparticles 45. In this case, AgCl formed *in situ* during the synthesis adsorbed on the seed crystal facets and facilitated the 3D anisotropic growth and formation of thorny gold nanostructures. The involvement of Ag^+^ ions in anisotropic growth was further verified by the X-ray photoelectron spectroscopy (XPS) analysis 45. It is also possible that poorly passivated defect sites and multiple twinning (crystal intergrowth or crystals sharing a facet) on the seed surface can play an important role in the anisotropic nanoparticle growth 46-48. Atta *et al.* showed that Ag^+^ ions play an important role in defining the crystallinity of the final nanostars and controls the number and length of thorns by directing the growth of the seed depending on seed twinning 49. This facilitated a highly reproducible production of nanostars with high monodispersity and plasmon tunability 49. While Ag^+^ ions direct the anisotropic growth by passivating the seed twinning, a recent study showed that thermal treatment can increase the crystal twinning in the seed nanoparticles, which resulted in high yield production of nanoparticles with preselected morphologies 50. This also provided an insight into the involvement of Ag^+^ ions in passivating the twinned facets in seed nanoparticles.

Silver is also known for its role as an underpotential deposition (UPD) agent, being able to direct the anisotropic growth of gold nanoparticles via surface passivation 51-54. In this mechanism, near-monolayers of Ag^0^ onto the growing nanoparticle surface can selectively adsorb on certain crystallographic facets, which restricts the growth of that crystal facet 40, 53. Even though various mechanisms explaining the role of silver in anisotropic crystal growth in plasmonic nanomaterials have been suggested, its precise role in the mechanism of the anisotropic crystal growth is still under debate.

### Good's buffers-based nanostar synthesis

**Figure 3:**
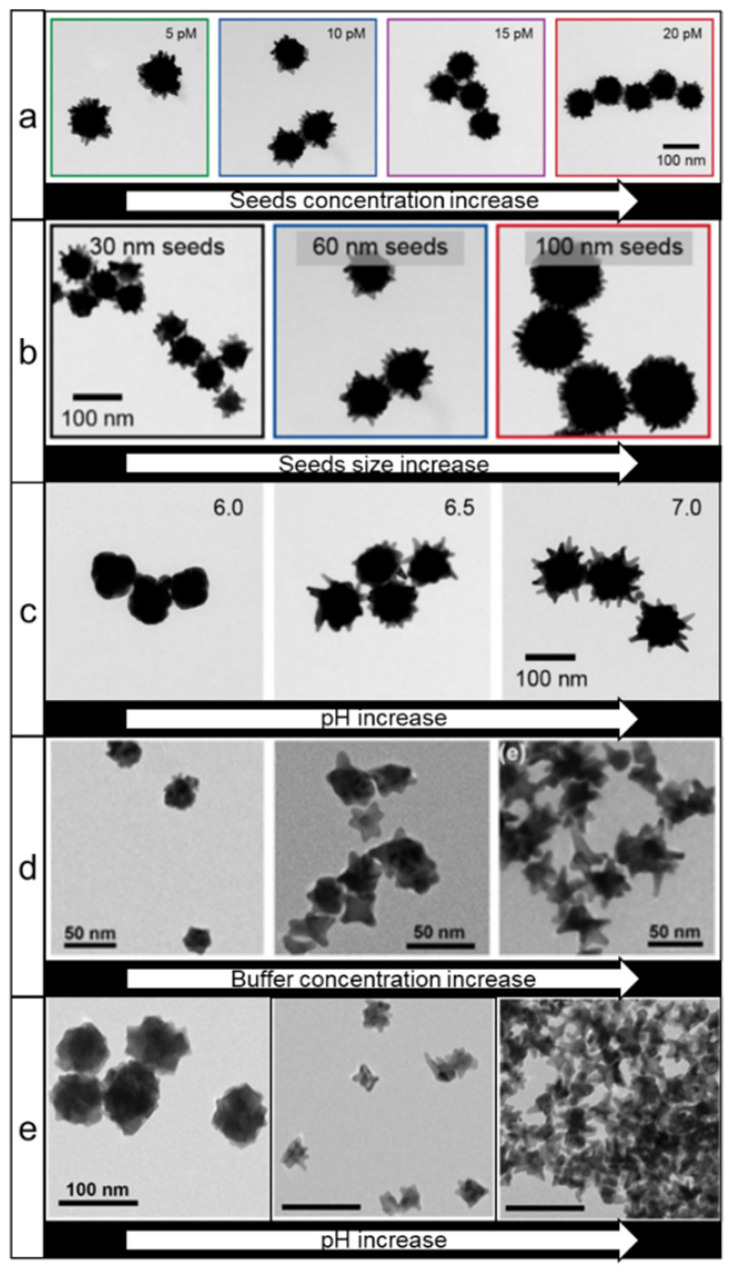
** TEM images of gold nanostars prepared using Good's buffers.** Effects of seeds concentration (a), size (b), and (c) pH increase on nanostars shape, adapted with permission from ref. 57, Copyright 2019 American Chemical Society. (d, e) Effects of buffer concentration and pH, respectively, on the nanostars size and shape during seedless synthesis, adapted with permission from ref. 59, Copyright 2014 American Chemical Society.

Good's buffers (e.g. HEPES, EPPS, MOPS, etc.) are generally zwitterionic water-soluble compounds with high biocompatibility and are widely used in biological research. The first report on the use of these buffers for nanostars synthesis dates back to 2005 55. It was demonstrated that nanostars could be produced with various shapes in these buffers by changing the pH and concentration of the Good's buffers. Xie *et al.* showed that the purity and homogeneity of the nanostars could be improved by controlling the temperature of the reaction 27. Interestingly, while the authors concluded that MOPS buffer did not promote the formation of nanostars, Odom *et al.* later reported that MOPS can be used for nanostars growth in both seedless and seed-mediated synthesis conditions 56, 57. The latter method is particularly interesting since it was demonstrated that by changing the concentration (Figure 3a) and size (Figure 3b) of the seeds, one can independently control the NPs core diameter and tip length. Furthermore, by varying pH, the sharpness of nanostar spikes can also be controlled (Figure 3c). An additional dimension of shape tuning was added by Pompa and coworkers, who utilized hydroxylamine as a reducing agent, while HEPES was mainly used for shape direction 58.

In contrast to seed-mediated methods, the seedless methods lack the ability to independently control sizes of core and tips without the use of auxiliary agents (Figure 3d, e) 56, 59. However, by adding some silver ions during a seedless synthesis, nanostar shape control can be achieved 60. A detailed investigation of the reaction mechanism showed that HEPES plays a dual role by binding to and stabilizing the surface of gold seeds via a complex, pH-dependent interplay between the nitrogen atoms and sulfonate groups, while reduction of gold ions onto the seeds occurs via oxidation of the nitrogen atoms to a cation radical as evidenced by electron paramagnetic resonance 61, 62.

### PVP-based nanostar synthesis

**Figure 4:**
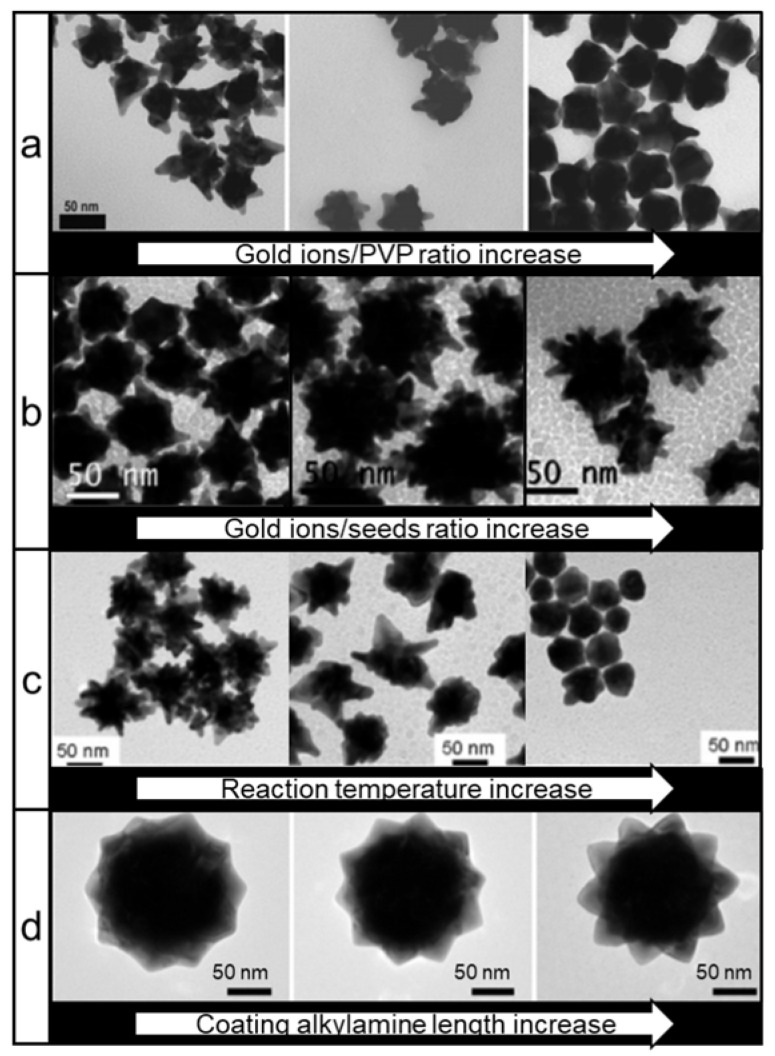
** TEM images of different types of gold nanostars synthesized via PVP-based route.** Nanostars shape control by changing: (a) gold ions/PVP ratio (adapted with permission from ref.^63^), (b) gold ions/gold seeds ratio (adapted with permission from ref.^64^, Copyright 2010 American Chemical Society, (c) reaction temperature during synthesis (adapted with permission from ref.^64^, Copyright 2010 American Chemical Society, (d) the alkyl chain length of an amine additive (adapted with permission from ref.^66^, Copyright 2015 American Chemical Society.

Another well-established method used for the synthesis of nanostars relies on the use of polyvinylpyrrolidone (PVP) as both a reducing and stabilizing agent. This seed-mediated method produces nanostars in high yields and with high monodispersity 63. Others demonstrated that the size, shape and thus LSPR of nanostars could be controlled by tuning the ratios of gold ions to gold seeds or by changing reaction temperature in the PVP/dimethylformamide (DMF) system (Figure 4a-c) 64, 65. An unprecedented degree of nanostar shape symmetry was achieved by the group of Lu who synthesized icosahedral gold seeds in diethyleneglycol (DEG)/PVP system and then grew nanostars from those seeds in a DMF/PVP system with the addition of various amines (Figure 4d) 66. Interestingly, this symmetry granted a noticeable increase of SERS enhancement in symmetric nanostars relative to their more asymmetric counterparts.

Although these protocols are CTAB-free and are capable of producing high yields of monodisperse nanostars, their reliance on DMF (a highly toxic organic solvent) for synthesis is an undesirable feature for biomedical imaging applications. Moreover, they demonstrate high adsorption of PVP to the gold surface, which in turn decreases the overall Raman signal intensity since the PVP will compete with the Raman reporters for the nanostar surface 67.

### Surfactant-free methods

**Figure 5:**
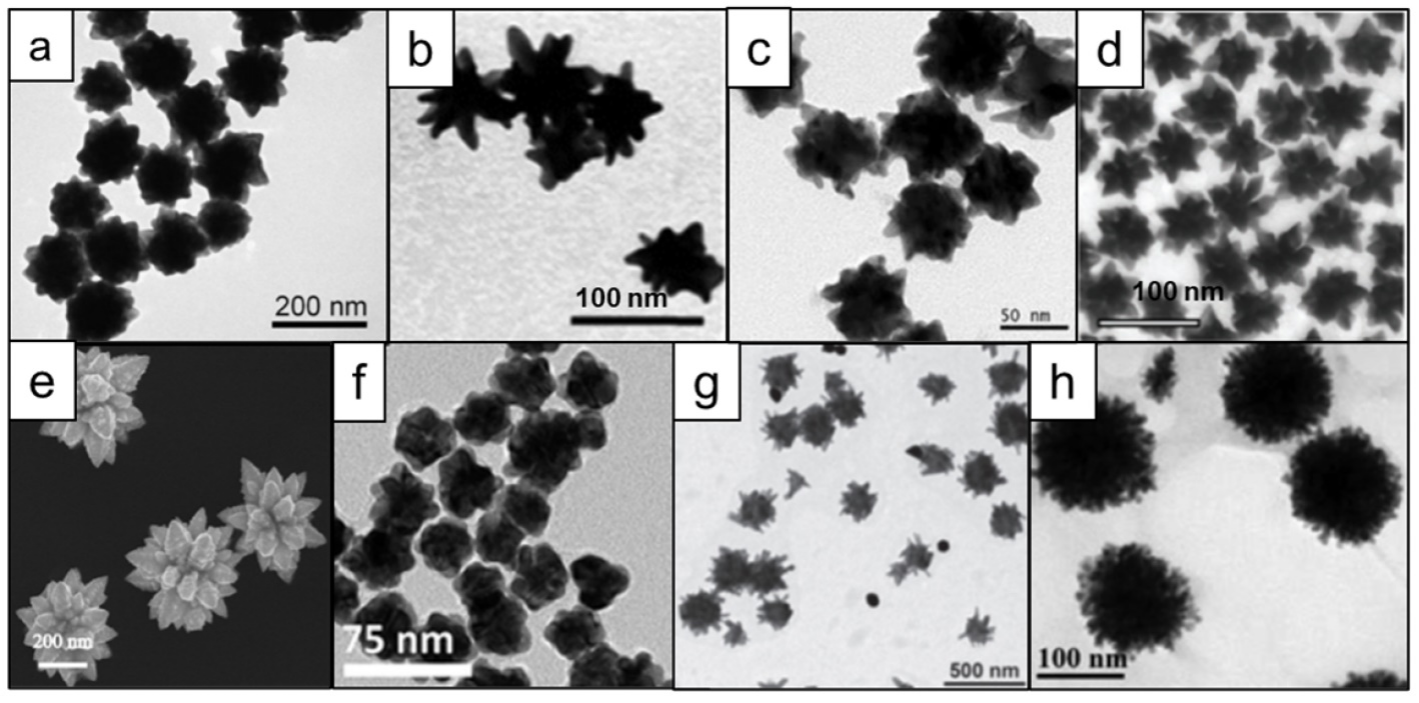
** Microscopy images of different shapes of nanostars that can be obtained in surfactant-free conditions using various reducing agents.** (a) hydroquinone, adapted with permission from ref. 68, Copyright 2011 American Chemical Society; (b) glucosamine, adapted from ref. 70 with permission from The Royal Society of Chemistry; (c) gallic acid 71, (d) hydrogen peroxide, adapted with permission from ref. 72, Copyright 2017 WILEY-VCH; (e) dopamine, adapted from ref. 73 under a Creative Commons Attribution (CC BY) license; (f) L-DOPA, adapted from ref. 74 with permission from The Royal Society of Chemistry; (g) tryptophan 75; (h) star fruit juice, adapted from ref. 76 with permission from The Royal Society of Chemistry.

In addition to the aforementioned synthesis procedures that either use toxic shape-directing surfactants or solvents (or combinations thereof), many efforts have been focused on designing syntheses that produce monodisperse nanostars in high yields under environmentally friendly conditions (*i.e.* aqueous conditions). Seed-mediated protocols using hydroquinone and citrate were reported in 2011, showing that pH and reagent concentrations determine the shapes of nanostars (Figure 5a) 68, 69. A seedless synthesis of nanostars that employed glucosamine or glucamine without any additional surfactants or shape-directing agents was reported by Moukarzel *et al.*, (Figure 5b) 70. Similarly, homogeneous nanostars were produced by using gallic acid (Figure 5c) 71. Kircher *et al.* developed a seed-mediated method to produce various shapes of nanoparticles using only alkaline hydrogen peroxide and gold chloride, demonstrating the capability to controllably generate various shapes and sizes of nanostars by closely tuning the reaction kinetics (Figure 5d) 72. In another report, ~400 nm star-like structures were obtained through the use of only dopamine as both the reducing and shape-directing agent 73 (Figure 5e). However, it was shown that by using a similar molecule, L-DOPA, one can also obtain nanostars of less than 100 nm size (Figure 5f) 74. Using an adduct of tryptophan and glutaraldehyde, Ma and colleagues have synthesized nanostars of several different shapes and relatively large sizes (>200 nm; Figure 5g) 75. Finally, star-like structures could also be obtained from solely star fruit juice and gold salt (Figure 5h) 76.

### Synthesis in microfluidic devices

To improve the reproducibility of nanoparticles synthesis in terms of size, dispersity and morphology, in recent years more research has been focused on the use of microfluidic technology for nanoparticle preparation instead of bulk syntheses 77-80. Microfluidic systems offer accurate control of various experimental parameters, such as sample volume, flow rate, mixing velocity, reaction time and temperature, providing a significantly more precise way to optimize the physical properties and hence imaging performance of SERS nanoprobes 78. As such, microfluidic systems with different flow profiles, specifically continuous and segmented (or droplet) flows, have been used to produce a range of plasmonic nanostructures including gold nanostars which have been used as plasmonic cores of SERS nanoprobes 80-85.

Continuous flow microfluidic systems have enabled the seedless synthesis and functionalization of gold nanostars with an average size of about 50-70 nm and a high aspect ratio 84. In a separate study, highly monodisperse gold nanostars averaging 60-80 nm in size were formulated using droplet microfluidics, either in the presence or absence of surfactants 85. In contrast to the continuous flow microfluidic system, the droplet microfluidic platform offers improved mixing due to chaotic advection, narrower sample distribution, and reduced cross-contamination. As a result, higher quality gold nanostars with more controlled properties can be produced using droplet microfluidics.

Altogether, further advances in microfluidic technologies are anticipated to enable the fabrication of more complex (*e.g,* coated instead of just the metallic core) SERS nanoprobes on a single microfluidic platform (instead of using multiple microfluidic devices), as well as synthesizing increasingly complex SERS nanostructures with precisely regulated physicochemical properties.

### Choice of Raman reporter

The choice of Raman reporter for a specific nanotag is as important as the choice of plasmonic core and depends on the surface properties of the metal nanoparticle core in terms of atomic composition (Au, Ag, Pt, etc.), zeta potential (positive or negative, absolute value), and the density of stabilizing agent. Independent of the plasmonic core, factors that are desirable for any Raman reporter include high Raman scattering cross-section (typically found in conjugated electron-rich polyenes or aromatic molecules like dyes); high photo- and chemical stability, molecular symmetry (to reduce the number of peaks in a Raman spectrum while increasing their relative intensity); absorption maximum in resonance with the excitation laser wavelength, and having anchoring groups with high affinity to the nanoparticle surface (*e.g.* thiols, disulfides, thiophenes, isothiocyanates, etc.) 86-89. Below we will discuss design considerations of Raman reporters to optimize Raman signal intensity of SE(R)RS nanotags.

### Aromatic thiols

Interest in aromatic thiols as Raman reporters arises from their ability to form strong gold-sulfur bonds,^90^ which leads to high gold surface coverage by such molecules 91. Popular examples of such reporters include 4-nitrothiophenol (4-NTP) 92, 93, 4-aminothiophenol (4-ATP)^93^, ^94^, and 4-mercaptobenzoic acid (4-MBA) 39, 95; all of which are simple aromatic thiols with minimal structural complexity leading to clean Raman spectra and potential for further conjugation.

**Figure 6:**
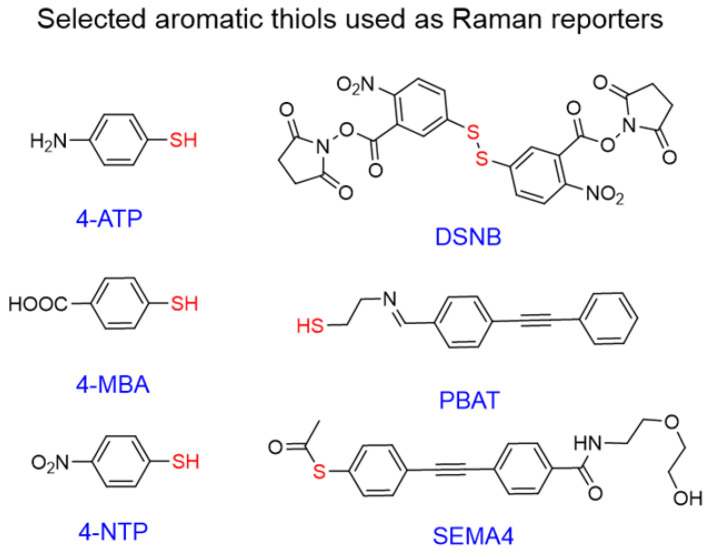
** Chemical structures of aromatic thiols used as Raman reporters in SERS nanotags**. Commonly used commercial reporters 4-NTP, 4-MBA, and 4-ATP and selected designer reporters SEMA4 96, PBAT^97^, and DSNB^98^. “Anchoring” sulfur atoms are shown in red.

Even though the vast majority of these molecules are commercially available, many “designer” Raman reporters have been synthesized to include additional functionalities (Figure 6). For example, small anchoring molecules containing triple bonds like SEMA4 or PBAT produce intense Raman scattering signal in the silent Raman region - a region found between 1750-2750 cm^-1^ where background signal from biomolecules is reduced 96, 97. DSNB was designed as a linker to attach antibodies to the gold surface, but also demonstrated favorable properties as a Raman reporter 98. Despite dense surface coverage, these molecules have absorption maxima in the 200-300 nm range and therefore are not in resonance with NIR lasers leading to reduced Raman scattering cross-sections.

### Organic dyes

Rhodamine 6G became very popular due to its large cross-section and has been widely used in SERS enhancement factor studies 6, 99, 100. Triarylmethine dyes, such as malachite green or crystal violet are also often used in SERS studies due to a combination of their large cross-section, negligibly low fluorescence and low price. Malachite green isothiocyanate is the most popular of the family due to the presence of the isothiocyanate anchoring group that provides higher affinity to the gold nanoparticle surface relative to other dyes 101, 102.

The most attractive Raman reporters for *in vivo* studies are NIR-absorbing dyes due to their resonant Raman signal enhancement when irradiated with a NIR laser. The majority of these dyes are based on cyanine 7 or 7.5 backbone, with different substituents granting tunability in absorption maxima position and Raman spectral fingerprints. Common examples of these dyes are IR 792 42, 103-105, IR 780p 106, 107, IR 780i 104, 108, 109, and DTTC 101, 103, 110, 111, which is often utilized as a benchmark dye for SERS intensity comparison studies 112.

Although multiple studies performed the comparison of SERS signal intensity of several commercial dyes, the Raman signal intensity is highly dependent on the type of plasmonic core and experimental conditions used, which complicates the interpretation of these results between different studies 103, 104, 113, 114. While some reports use the Raman signal intensity of a common chemical such as ethanol 103 as a benchmark (e.g. SERS nanotags made with dye A produce X times higher Raman signal than ethanol, while the same nanotags made with dye B produce Y times higher signal), currently there is no universal standardized protocol for reporting SERS signal enhancements that enables the uniform comparison of signal intensities between different groups.

### Rational Raman Reporter Design

**Figure 8:**
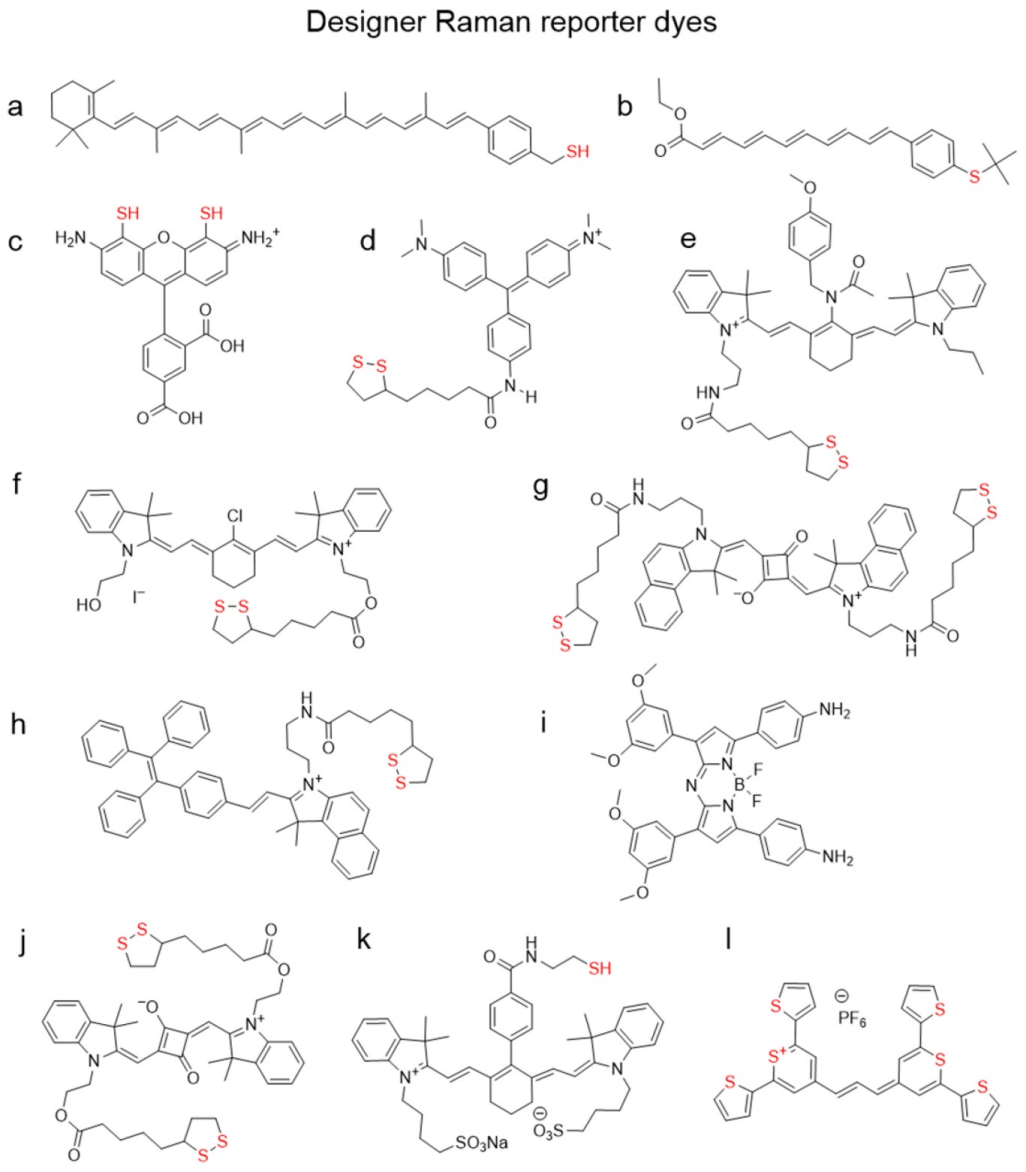
** Chemical structures of organic dyes designed to be reporters for SERS nanotags.** (a) Carotenethiol^115^, (b) polyenethiol^116^, (c) thiolated rhodamine^117^, (d) B2LA^118^, (e) CyNAMLA-381^112^, (f) Cy7-lip^119^, (g) SQ3^120^, (h) TPE-In-L^121^, (i) aza-BODIPY^122^, (j) M1^123^, (k) IR783B^124^, (l) chalcogenopyrylium dye^127^. “Anchoring” sulfur atoms are shown in red.

Raman reporters often require specific design characteristics that are not found in commercially available dyes. As such, a lot of efforts have been focused on the design of Raman reporters that have a high affinity for the gold nanoparticle surface and are resonant with the excitation laser. For example, by adding a thiol group to β-carotene (Figure 8a), Kneipp *et al.* demonstrated a 9-fold increase in SERS signal in comparison to the parent molecule 115. Similar polyene molecules were synthesized by Keller *et al.* (Figure 8b) 116. Adding thiol groups to the xanthene ring of a rhodamine in an attempt to increase its anchoring abilities has been reported, but the designer dye CRh-SH (Figure 8c) failed to outperform commercial rhodamine 6G 117. The group of Chang published a library of triphenylmethine dyes containing lipoic acid residue, with the brightest dye being B2LA (Figure 8d)^118^. However, since that family of dyes was not in resonance with biologically relevant 785 nm laser, they later developed 80 more dyes derivatized with lipoic acid 112. In this instance the dye scaffold was based on the NIR dye cyanine 7. Their dye CyNAMLA‐381 (Figure 8e) produced a 12 times higher Raman intensity relative to the DTTC under similar experimental conditions. A cyanine 7-lipoic acid conjugate (Cy7-lip; Figure 8f) was shown to outperform conventional rhodamine 6G by more than 10 times 119. A similar approach of adding a lipoic acid residue for anchoring the dye to the gold nanoparticle surface was demonstrated by the group of Maiti with a squaraine dye and a tetraphenyl ethylene (TPE) derivative (Figure 8g, h) 120, 121. Another dye developed by the same group belongs to a NIR family of aza-BODIPY dyes (Figure 8i) and was reported to have significantly higher Raman intensity than crystal violet or rhodamine B 122. The bidentate lipoic acid-conjugated squaraine dye M1 (Figure 8j), developed by the group of Liu, was shown to permit the selective growth of silver nanospheres on the tips of gold nanostars 123. IR783B, reported by Yue *et al.*, is a cyanine dye containing a thiol moiety for anchoring purposes and is resonant with 785 nm laser (Figure 8k) 124. A more unconventional approach to a NIR Raman reporter design was chosen in a collaborative effort between the groups of Kircher and Detty, who, instead of adding a sulfur-containing linker to an existing dye, synthesized chalcogenopyrylium-based chromophores containing sulfur in the dye core (Figure 8l). The corresponding SERRS nanotags demonstrated the highest Raman signal sensitivity to date, which was in the attomolar range 12. Furthermore, this Raman reporter type can be further red-shifted by substituting the sulfur for selenium atoms and/or extending the conjugation chain to allow these dyes to be resonant with even longer wavelength excitation lasers such as 850 and even 1064 nm 125, 126.

## Coating types

To protect the metal-molecule system from environmental factors that could lead to decreased colloidal stability and/or desorption of Raman reporters from the metal core, it is embedded in an encapsulant layer. The most commonly used encapsulants include silica, synthetic polymers, and biomolecules such as proteins or lipids, and sometimes a composite shell can include a combination of these components.

### Polymer coating

Polymers have been widely used as stabilizing agents. They allow for a high degree of flexibility, because their properties that include biodegradability rate, hydrophobicity, or polymer chain packing, can be finely tuned by altering their chemical structure and ultimately dictate physicochemical properties of the SERS nanotags 128.

The most widely used polymer in the fabrication of nanoparticles for biomedical applications is polyethylene glycol (PEG) - it is highly hydrophilic, chemically inert, and has antifouling properties to prevent opsonization of biomolecules on the nanoparticle surface, providing “stealth-like” properties leading to increased circulation times 129, 130. Nie *et al.* were the first to produce injectable SERS nanotags that constituted spherical gold nanoparticles coated with Raman reporters that were stabilized by thiolated-PEGs 101. This PEG layer can be attached both to the gold surface directly 95, 101, 131 and to another coating layer 132, 133.

Polydopamine (PDA) is another biocompatible polymer used to stabilize gold nanoparticles 134, 135. It is formed *in situ* during nanoparticle surface coating with molecular dopamine. Due to its high affinity towards calcium ions, PDA was used to create SERS nanotags which specifically targeted bone microfractures after either intravenous or intramuscular injection 136.

Amphiphilic polymers have been utilized extensively in NPs coating strategies due to their ability to adsorb onto hydrophobic surfaces and render them hydrophilic 137. Such properties of the amide conjugate of polyisobutylene-alt-maleic anhydride (PMA) and dodecylamine were highlighted by Liz-Marzán, where the polymer was used for the coating of SERS nanotags with a high surface coverage of hydrophobic Raman reporters 138. As was demonstrated by Chen *et al.*, amphiphilic copolymers (such as polystyrene-block-poly(acrylic acid) in this instance) are compatible not only with various Raman reporters but also with nanoparticles of different shapes and sizes 139.

**Figure 9:**
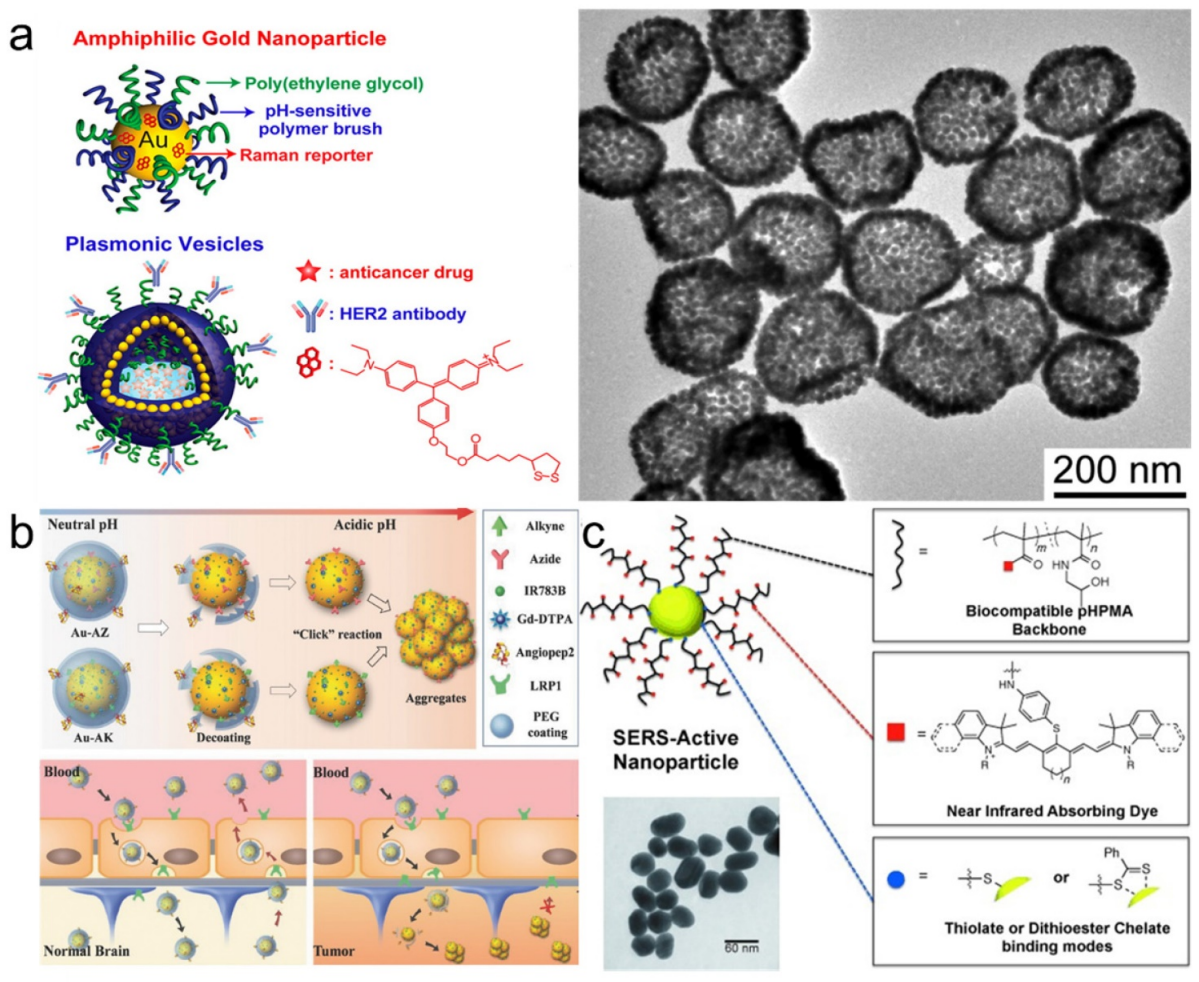
** Polymer-coated SERS nanotags.** (a) Schematic illustration (left) and TEM image (right) of plasmonic vesicles self-assembled from dual polymer-coated SERS nanotags, adapted with permission from ref. 140, Copyright 2010 American Chemical Society; (b) pH-responsive PEG-coated nanotags which self-assemble inside brain tumors, adapted with permission from ref. 141, Copyright 2017 WILEY-VCH; (c) SERS nanotags with a dye-conjugated polymer coating for dual SERS-fluorescence imaging of lymph nodes, adapted with permission from ref. 143, Copyright 2014 WILEY-VCH.

Combining amphiphilicity with pH-sensitivity of coating polymers allowed Song *et al.* to create plasmonic vesicles made entirely of self-assembled gold nanoparticles that incorporated a high number of hotspots 140. First, 14 nm gold NPs were independently coated with both hydrophilic PEG chains and a hydrophobic copolymer of 4-vinylpyridine (4VP) and methyl methacrylate (MMA). Then these small nanoparticles were self-assembled into 100-200 nm vesicles which were internalized by cells and disassembled in lysosomes specifically due to the pH-sensitive nature of their hydrophobic copolymer coating component (Figure 9a). Moreover, attaching PEG to the gold surface via the acid-responsive hydrazone linker allowed the group of Li to develop SERRS nanotags that could cross the blood-brain barrier and then self-assemble inside tumors to create hotspots and dramatically enhance their Raman signal (Figure 9b) 141.

Since polymers compete with the Raman reporter for the gold nanoparticle surface, this can lead to uneven or diminished loading of the Raman reporter, resulting in decreased Raman signal. To address this issue, NIR-absorbing polymers, such as conducting polymers polyaniline or polypyrrole, were used as Raman reporters. Liu *et al.* demonstrated that these polymers coated onto gold nanorods exhibit strong resonant Raman scattering enhancement when irradiated with biologically relevant 785 nm laser 142. Another way to combine reporter and coating was demonstrated by Iacono *et al.*, who synthesized a thiol-terminated poly(N‐(2‐hydroxypropyl)methacrylamide and covalently conjugated NIR dyes throughout the polymer chain (Figure 9c)^143^. This NIR reporter-containing coating not only stabilized the NPs, but also allowed to perform both Raman and fluorescence imaging to detect lymph nodes.

Microgel-forming and thermoresponsive poly(N-isopropylacrylamide) (pNIPAM) was used as a coating , but in this instance Raman reporters were added after polymer attachment - due to its porous nature, reporters could diffuse inside the microgel and reach the gold surface 144. Subsequently, the pores were “sealed” through a layer-by-layer assembly of poly(allylamine hydrochloride) and poly(acrylic acid). The thermo-responsive nature of pNIPAM was highlighted by Kearns *et al.* who created a hollow nanogold-based SERS nanotag coated with pNIPAM having a Raman reporter on the distal end of the polymer 145. Such nanotags changed the SERS signal intensity upon laser irradiation due to photothermal heating, because pNIPAM brought the reporter closer to the gold surface upon collapsing. A similar approach, but with a thermo-responsive copolymer of pNIPAM and acrylamide was utilized by Song *et al.* to coat branched gold nanoshells 146. Upon heating to 40 °C this polymer collapsed, which was shown by the increase in the rhodamine B Raman scattering that was attached to it. Such response to heating was used to load the nanoshells with drugs and subsequently release them upon laser irradiation.

Even though stability of nanotags during storage is a valid concern, using polystyrene for coating allowed Yu *et al.* to create extra stable nanotags with minimal SERS signal loss upon storage up to half a year in solution 147.

### Silica coating

Primary reasons for the popularity of silica as an encapsulating layer include chemical inertness, mechanical rigidity, biodegradability, lack of contribution to the nanotag Raman spectrum and high degree of tunability 148, 149. Shell thickness, porosity, chemical stability and biodegradability can be tuned depending on the desired application. However, not all silication protocols are suitable for yielding high intensity SERS nanotags, and below we will discuss the evolution of strategies used to develop protocols for the brightest nanotags.

**Figure 10:**
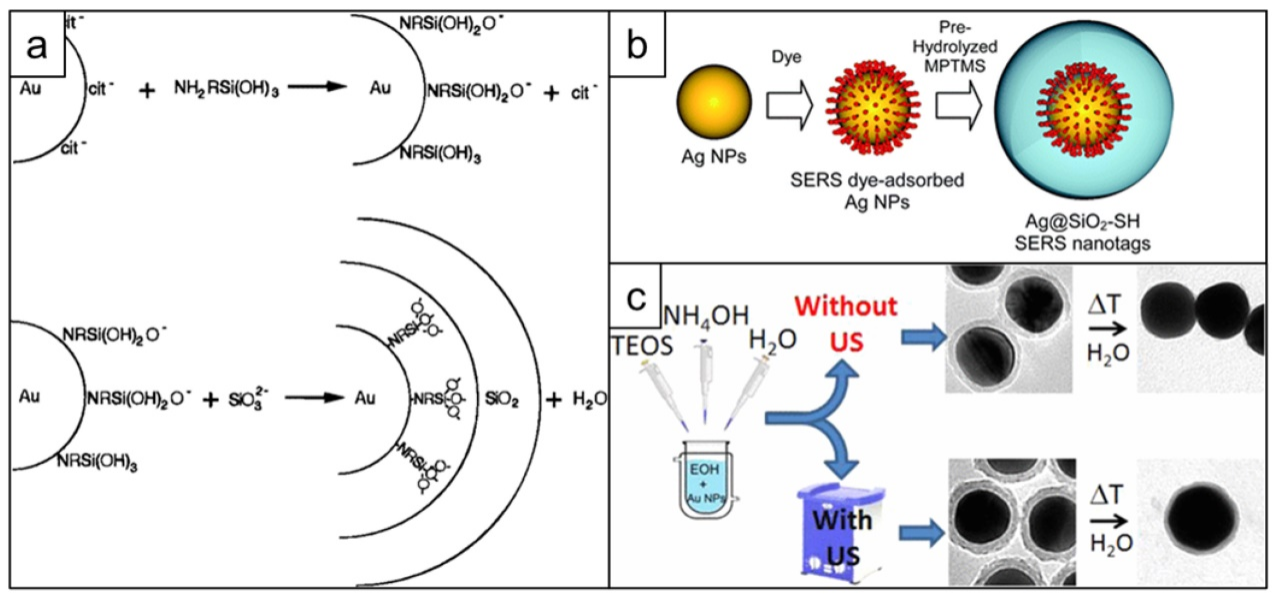
** Development of silica coating for SERS nanotags**. (a) Schematic representation of the surface reactions taking place during first successful two-step silica coating of gold nanoparticles, adapted with permission from ref. 151, Copyright 1996 American Chemical Society; (b) Scheme showing one step silication of a SERS nanotag, adapted from ref. 154 with permission from The Royal Society of Chemistry; (c) Stabilizing effect of applying sonication during nanotag silication, adapted with permission from ref. 160, Copyright 1996 American Chemical Society.

The first report of silica coating of gold nanoparticles dates back to 1996 and describes a multi-step process, which includes: 1) gold nanoparticle surface priming with (3-aminopropyl)trimethoxysilane (APTMS) or (3-mercaptopropyl)trimethoxysilane (MPTMS), 2) hydrolyzing the APTMS/MPTMS layer in the presence of sodium silicate to create a thin (2-4 nm) silica shell over 24 h and finally 3) growing thicker silica shell using classical Stӧber method 150 - hydrolysis of tetraethyl orthosilicate (TEOS) by ammonia in aqueous ethanol (Figure 10a) 151, 152. Later this protocol was adapted for synthesizing SERS nanotags by adding the dropwise addition of the Raman reporter between steps 2 and 3 153. Considering that both the surface primer and the Raman reporter compete for the gold nanoparticle surface, it was important to find an optimal ratio that enabled the maximum amount of Raman reporter to adsorb to the gold nanoparticle surface whilst ensuing enough primer was present to render the surface vitreophilic. Even though earlier reports required the Raman reporter dye to have an anchoring group, such as isothiocyanate 152, this requirement was later rectified 154 (Figure 10b). Alternatively, surface priming is performed with PVP from which the silica shell is grown using a Stӧber method 155. To improve surface coverage of the Raman reporter, a consecutive coverage with a monolayer of the high affinity Raman reporter DTNB, polyallylamine and PVP was performed by Schlücker and co-workers before growing the silica shell 156. The same group also demonstrated that a conjugate of APTMS and mercaptobenzoic acid acted both as a reporter and a primer for silica growth 157. Typically, silication is performed in alcohols, which destabilizes the nanoparticles leading to aggregation. To increase nanoparticle stability against precipitation in ethanol during the silication process, Mir-Simon *et al.* utilized mercaptoundecanoic acid as a surface primer and demonstrated it was less detrimental to the Raman reporter signal than PVP 158. Similarly, Fales *et al.* stabilized gold nanoparticles with thiol-PEG to prevent aggregation of the gold nanostars during subsequent silica coating in the presence of DTTC 159. A drawback of using capping agents such as thiol-PEG is that these agents have a high affinity for the gold nanostar surface and outcompete low affinity Raman reporters such as DTTC.

Ideally, a primer-free silication method that is compatible with a wide variety of Raman reporters would be used. A protocol fulfilling this need was published by the Kircher group. A primer-free synthesis protocol of SERRS nanostars was developed, which, due to the lack of competition for the nanoparticle surface, produces unprecedented SERRS signals 13, 26, 127. Others demonstrated that ultrasonication could be used during the synthesis of silica shells to significantly improve their stability towards hydrolysis (Figure 10c) 160.

### Biomolecular coating

#### Proteins

Bare metallic nanoparticles coming into contact with the biological environment are known to adsorb surrounding proteins, forming what is called a “protein corona” 161, 162. However, selective precoating of the NPs with proteins allows more control over the protein corona composition, and as a result, nanoparticle properties 163. One of the most popular choices for this approach is bovine serum albumin (BSA). Coating of the nanotag surface with BSA is a rapid and straightforward approach to enhance the colloidal stability and biocompatibility of the nanotags. BSA was used, for example, in the preparation of Raman-based pH sensors 164 and dual SERS-upconversion fluorescence nanotags 165. Glutaraldehyde-crosslinked BSA was utilized by Samanta *et al.* as a more stable version of BSA coating for their series of SERS nanotags 112. Moreover, there are reports showing that BSA coating can prevent NP aggregation more effectively than PEG coating 104. As shown by Blanco-Covián *et al.*, antibodies can also be used as a stabilizing layer when they are attached directly to the gold surface 166.

#### Nucleic acids

Owing to their high hydrophilicity, DNA aptamers can be used not only for targeting but also as a stabilizing coating layer. For example, in a report by Wang *et al.* the authors directly attached thiol-terminated DNA aptamers to the surface of gold nanoparticles, which allowed them to develop an apta-immunoassay for cancer exosomes detection 167. Another assay was developed for prostate-specific antigen detection by Jiang and coworkers based on DNA-covered SERS nanotags 168. Using SERS nanotags with polycytosine Li *et al.* created a sensor which increases its Raman signal intensity upon Hg^2+^-induced nanotag aggregation 169. Different thiol-terminated DNA sequences were also used by Tang and coworkers to coat SERS nanotags for the quantification of miRNA in living cells 170. Another application of DNA-coated SERRS tags was demonstrated by Simoncelli *et al.* who developed optothermally tunable plasmonic nanoantennas capable of single-molecule detection 171.

#### Lipids

The main advantages of lipids as coatings include their inherent biocompatibility and highly predictable nature of self-assembly based on the composition of lipid mixture. The first lipid-encapsulated SERS nanotags were developed in 2010 by the Zheng group 172. They showed that by using a mixture of single- and double-chain phospholipids one could encapsulate Raman reporter-coated gold nanoparticles inside lipid bilayer without the use of sulfur-containing surface primers and losing Raman signal intensity. Later on, Walker and colleagues published their protocol for the synthesis of SERS nanotags with a lipid coating, which utilized a different lipid bilayer composition and was compatible with three different types of Raman reporters 173. The same group used the developed phospholipid coating to demonstrate controlled tip-to-tip aggregation of gold nanorods, creating strong hotspot SERS enhancement in the junction 174. Su *et al.* 175 exploited the dynamic nature of lipid bilayers to demonstrate the environmental sensitivity of their SERS nanotags. In this instance, depending on the Raman reporter used, the Raman signal changed in response to heating, presence of other lipid bilayers or the presence of surfactants. A lipid-porphyrin conjugate coating was developed by the group of Zheng,^176^ which combined extra high loading of the Raman reporter inside lipid bilayer with stabilization it provides. A relatively unconventional synthetic approach was chosen by the group of Kang,^177^ who instead of coating gold NPs with lipids, synthesized the gold nanoparticles inside pre-made liposomes.

## Nanotag targeting for *in vivo* imaging

Raman imaging using SE(R)RS nanostars offers highly sensitive and specific disease detection. Their narrow, fingerprint-like spectra make SE(R)RS nanotags ideally suited for *in vivo* multiplexed molecular imaging using up to 10 different 'flavors' of SERS nanoparticles 178*.* Since the Raman reporters can be selected to generate a unique Raman fingerprint that is not found anywhere else in the biological system, unprecedented signal to background ratios can be obtained.

The Kircher group demonstrated that due to the femtomolar detection sensitivity of SE(R)RS nanostars, it was possible to image bulk tumor, microscopic infiltration into normal surrounding tissues, residual tumor after surgical resection, and even micrometastases in animal models that closely recapitulate pancreatic, prostate, breast, and colon carcinogenesis, among others, after intravenous administration 26, 132. Non-targeted nanoparticles typically accumulate in tumor tissue via leaky neovasculature and are retained due to reduced clearance by a locally dysfunctional lymphatic system as well as increased influx of phagocytic cells - a phenomenon collectively known as the enhanced permeability and retention (EPR) effect 179-181.

While non-targeted nanoparticles have been shown to be highly effective in delineating solid tumors irrespective of their site of origin, no information is provided on the (molecular) composition of the tumor. To address this, targeted approaches using SE(R)RS nanoparticles were explored. The first demonstration of targeted SERS nanoparticles was performed by Nie and coworkers in 2008 who used thiol-PEG stabilized SERS nanoparticles that were linked to epidermal growth factor receptor (EGFR) targeting antibodies 101. They showed that EGFR-targeting improved the tumor uptake of the SERS nanotags by 10-fold relative to their non-targeted counterparts. Using a silica-coated gold nanostar core, Nayak *et al.* showed that tissue factor-targeted SERRS nanotags could visualize breast cancer metastases in the lungs, while non-targeted nanotags failed to do so under the same experimental conditions 182. Similarly, Oseledchyk *et al.* created a cocktail of folate receptor-targeted and non-targeted SERS nanostars that could visualize folate receptor-expressing ovarian cancer metastases using a ratiometric imaging approach 106. In another application, SERS nanostars were used for simultaneous detection of EGFR and programmed death-ligand 1 (PD-L1) in breast cancers using multiplexed detection of EGFR- and PD-L1-targeting antibody-labeled nanotags, respectively 183. In a follow-up study, the same group utilized a combination of PD-L1- and CD8-targeted SERRS nanostars to that accurately detect PD-L1+ tumor cells and CD8+ T cells simultaneously *in vivo*. Moreover, this combination allowed the monitoring of treatment response, i.e. CD8 T cell recruitment to the tumor, following PD-L1 immunotherapy 184. Lastly, Wang *et al.* used a cocktail of 3 different SERS nanostars. Their Raman reporters consisted of bioorthogonal molecules that embedded a combination of alkyne, azido, and cyano- functionality to generate a Raman fingerprint in the Raman silent region 185 - the spectral window between 1,750-2,750 where biological molecules typically do not emit any Raman signal. The reporters for the Raman silent region were subsequently encapsulated in silica, and targeting moieties were conjugated to the nanoparticle surface via heterobifunctional PEG linkers. The targeting moieties consisted of a nucleolin-targeting aptamer AS1411, an integrin α_v_β_3_-targeting cyclic RGD peptide, or a CD44 antibody. Intravenous injection of this cocktail of targeted SERS nanostars allowed for multiplex phenotyping of breast cancer in animals bearing two MDA-MB-231 and MCF-7 tumors. Furthermore, this group confirmed that targeting improved tumor uptake 10-fold. In other studies, receptor-targeted SERS nanostars (*e.g.* integrin α_v_β_3_ and prostate-specific membrane antigen (PSMA)) enabled super-resolution SERS imaging to probe membrane receptor interactions in cells 186, 187, providing chemical information and spatial resolution with potential for diverse applications in life science and biomedicine such as for instance lateral flow assays for early (multiplexed) disease marker detection in bodily fluids 188-190.

The aforementioned studies demonstrate how the high signal sensitivity and specificity provided by SE(R)RS nanostars enable highly precise cancer imaging as well *in vivo* molecular imaging to allow for immediate tumor phenotyping as well as tumor monitoring.

## Multimodal imaging and theranostic applications

Owing to their high uptake by tumors and high signal specificity, SE(R)RS nanostars offer tremendous opportunities for cancer diagnostics and imaging 191, 192. However, like any other optical imaging technology, tissue depth penetration is limited and therefore deep tissue- or whole-body imaging is not supported by conventional Raman imaging. To improve detection of SERS nanotags at tissue depths that go beyond conventional Raman imaging detection, spatially offset Raman spectroscopy (SORS) or surface-enhanced spatially offset resonance Raman spectroscopy (SESORRS) can be used 42. We recently discussed this up-and-coming technology in-depth 193. Another approach would be to combine Raman imaging with magnetic resonance imaging (MRI), positron emission tomography (PET), fluorescence imaging and computed tomography (CT). The nanoparticle surface can be modified to incorporate metal chelating functional groups to chelate, for example, gadolinium or manganese to enable MRI functionality, or radioisotopes to render the nanoparticle PET-active 184, 194. In the case of silica-encapsulated SE(R)RS nanotags, the silica itself has high affinity for oxophilic cations such as gadolinium (MRI) or zirconium-89 (PET) and obviates the need for introduction of chelating groups 108, 195. Less oxophilic (radio)isotopes such as copper-64 can be sequestered via thiolation of the SERS nanotag's silica surface using 3-mercaptopropyltrimethoxysilane (MPTMS) to produce multimodal PET-active SERS nanoparticles 196. SERS nanotags that have gold cores provide intrinsic contrast on CT and could be used in a whole-body imaging setting 197. Moreover, due to strong near-infrared absorption gold nanostars are particularly well-suited as contrast agents for photoacoustic (PA) imaging and SERRS-active gold nanostars enabled dual-modal Raman/PA imaging of glioblastoma 107. The photoacoustic effect is achieved through rapid absorption of pulsed laser energy by the gold nanostar and its conversion to heat which causes a transient thermoplastic expansion of the gold nanostar core, that generates an ultrasonic band that can be detected using an ultrasound receiver. If instead of a pulsed laser, a high-power (>100mW) continuous laser is used that matches the LSPR of the gold nanostars, the heating is not transient and can be used to heat up the local microenvironment surrounding the gold nanostars in a process known as the photothermal effect. In turn, this can be used to destroy tumor cells in a procedure called photothermal therapy (PTT), applicable to various cancer cells. SERS nanostars were used by Liu *et al.* to completely ablate the tumor using PTT after only 20 minutes of irradiation with no signs of recurrence after a week 198. Another example of complete tumor ablation using nanostars was demonstrated by Gao *et al.*, but this time after 30 minutes of laser irradiation 199. Moreover, SERS nanotags used for PTT can be loaded with anticancer drugs for chemotherapy 200, 201, light-activatable molecules for photodynamic therapy (PDT) 109, 159 or release toxic and generate reactive oxygen species 202, 203.

## Conclusions and outlook

In recent years, SERS nanotags have evolved from rather simple nanoparticles used primarily for *in vitro* assays to complex multicomponent nanoconstructs tailored for use in biomedical diagnostic and therapeutic applications. Even though the principles of the design of SERS nanotags have already been outlined, the development of every aspect of a SERS nanotag is a subject of ongoing research and improvement.

Significant progress has been made in diversifying anisotropic metal core shapes and their respective synthesis protocols, allowing researchers to finely tune the final shape, size and surface properties of nanostars and reach enhancement factors unattainable with spherical nanoparticles. However, preparation of narrowly dispersed nanoparticle preparations remains a challenge and, consequently, there is a need for reproducible synthesis procedures to obtain nanoparticles with structural homogeneity with pristine surfaces; continuous in-line microfluidics-based synthesis approaches could provide a potential solution. While the general design principles of Raman reporter molecules can be considered established (*i.e.* high Raman cross-section, resonance with laser, high affinity for plasmonic core surface), to date only a limited amount of dye-scaffolds has been used and the most widely-used resonant Raman reporters are based on commercial cyanine dyes. Development of novel dye designs would greatly benefit the multiplex capabilities of SE(R)RS-based biomedical strategies. As discussed, SERS nanotag coating is the most diverse component of the construct as it not only is involved in nanoparticle stabilization, but also acts as a base for targeting moiety conjugation, as well as (intrinsic) chelation of medical isotopes to produce targeted-SERS nanotags with multimodal capabilities.

Preclinical evaluation of gold nanostar-based SERS nanotags have shown the tremendous promise of such agents for highly sensitive disease (marker) detection and even therapeutic intervention using PTT. However, upon intravenous administration, SERS nanotags, like any nanoparticle of similar size or composition, not only accumulate in tumor tissues, but also in organs of the reticuloendothelial system (RES) such as liver and spleen. Although gold is metabolically inert and silica-based coatings are fully biodegradable, the effect of long-term retention on these organs remains unclear. Therefore, efforts are being directed to designing truly biodegradable or excretable SERS nanotags that retain their high signal intensity.

Lastly, design of medical-grade Raman imaging systems are required for the clinical translation of contrast-enhanced Raman imaging approaches. We recently demonstrated for the first time that a clinical Raman endoscope 204 enabled the detection of incipient cancers in the gastrointestinal tract following SERRS nanoparticle administration in preclinical models of colorectal carcinogenesis 132. Similarly, a wide-field Raman imaging system could enable Raman-guided tumor resection in cancer patients. While such systems are currently being developed 205, they currently do not enable real-time imaging due to the relatively long acquisition times/area relative to fluorescence imaging. However, rapid advancements in optical technologies and new insights in Raman instrument design may someday lead to the development of a real-time, wide-field Raman imaging system that will enable Raman-guided macroscopic tumor resection and photothermal ablation of residual microscopic tumor cells in the resection bed when paired with intravenously administered SERRS nanostars; a truly nanotheranostic platform.

## Figures and Tables

**Figure 7:**
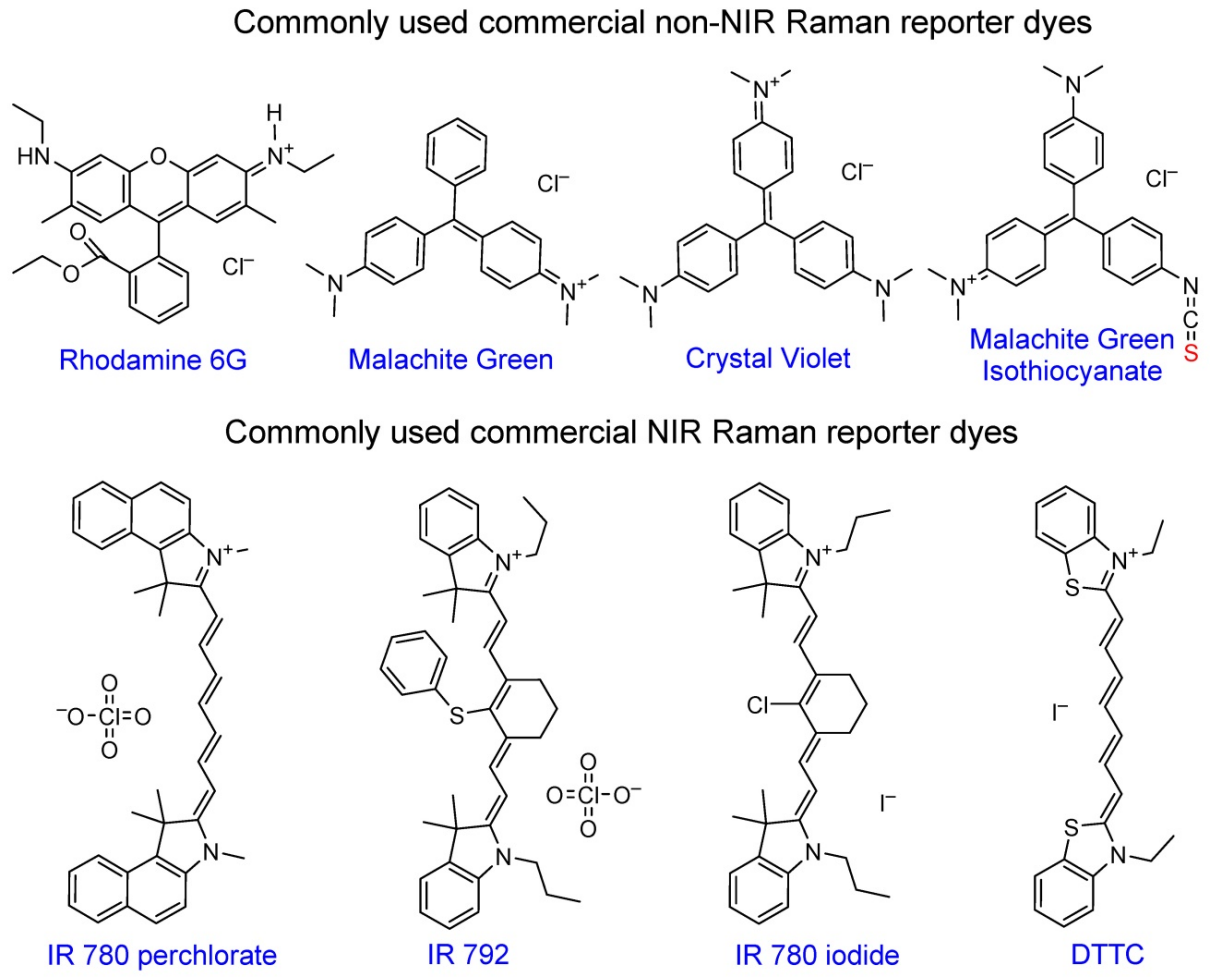
** Chemical structures of popular commercial organic dyes used as Raman reporters in SERS nanotags.** “Anchoring” sulfur atoms are shown in red.

**Table 1:** Selected examples of gold nanostars synthesis methods.

Reducing agent	Auxiliary shape-directing or capping agent	Seeds presence	Comments	Reference
Ascorbic acid	CTAB, Ag^+^	Seed-mediated	First example of nanostars synthesis, requires toxic CTAB.	30
Ascorbic acid	Ag^+^	Seed-mediated	Surfactantless nanostar synthesis allowing to obtain higher SERS signal.	37
Ascorbic acid	-	Seedless	Surfactantless, silver-free and seedless nanostar synthesis allowing to obtain highest SERS signal.	13, 26
Ascorbic acid	-	Seed-mediated	Surfactantless, silver-free and seed-mediated method of larger scale nanostar synthesis, allowing to obtain highest SERS signal.	42
H_2_O_2_	-	Seed-mediated	Surfactantless, silver-free and seed-mediated method that enabled shape-direction by tuning of the reaction kinetics using pH and H_2_O_2_	72
Good's buffers	-	Seedless	High tip/core ratio, high polydispersity of obtained nanostars.	56
Good's buffers	-	Seed-mediated	Tip/core ratio and nanostars sizes can be controlled independently, high size and shape monodispersity of the obtained nanostars.	57
PVP	-	Seed-mediated	High monodispersity and shape/size control, however method requires toxic DMF as a solvent.	64
PVP	-	Seed-mediated	Highest known monodispersity and shape control of obtained nanostars grown from monodisperse symmetrical seeds. Provide significantly higher SERS signal than asymmetric nanostars.	66

**Table 2:** Selected examples of SERS nanotags coatings.

Coating type	Comments	Reference
PEG	First example of successful *in vivo* tumor imaging using SERS nanotags covered by PEG layer attached directly to gold surface.	101
PEG, silica	While silica coating prevents Raman reporter from detaching from gold surface, PEG layer increases nanoparticles blood circulation time and decreases liver and spleen uptake.	12, 13, 26, 132
PDA	PDA coating not only granted SERS nanotags biocompatibility, but also allowed bone microfractures imaging due to high affinity of PDA towards calcium ions.	136
PMA-dodecylamine amide	Amphiphilic nature of the coating polymer allowed nanotags with high surface coverage by hydrophobic Raman reporters become hydrophilic and colloidally stable in water.	138
PEG, PMMA-*co*-4VP	Due to pH-sensitive nature of PMMA-co-4VP coating polymer, the multinanoparticle liposomal SERS construct could be disassembled in lysosomes following cellular uptake, allowing effective monitoring and controlled release of cargo.	140
Polyaniline Polypyrrole / PVP	Conjugated polymers for nanoparticles coating used in the study provided a viable alternative to organic dyes as SERS reporters, however they required additional coating with PVP to increase colloidal stability.	142
PNIPAm-*co*-AAm PEG	By coating drug-loaded branched gold nanoshells with thermoresponsive PNIPAm-*co*-AAm with a Raman reporter at a terminal end, it was possible to monitor photothermal heating and drug release from the nanoparticles by increase in SERS signal due to polymer collapse.	146
Polystyrene	Remarkably stable polystyrene coating for SERS nanotags allowed their storage for 6 months without significant loss of SERS intensity.	147
Silica	First primer-free silica coating protocol that allowed to achieve unprecedentedly high SERS intensities due to absence of competition for surface for the Raman reporter.	26
Silica	Ultrasound-assisted silica coating of nanoparticles can increase the shell stability towards hydrolysis.	160
BSA	BSA coating was shown to better protect gold nanostars from reshaping into spheres and aggregation than PEG coating after storage for 24 hours at room temperature.	104
DNA	Coating SERS nanotags with oligonucleotides was demonstrated to not only increase the particles colloidal stability, but also allowed simultaneous imaging and quantification of multiple miRNAs inside cells in vitro.	170
Lipids	Lipid membrane coating of gold nanoparticles allows encapsulation of multitude of different Raman reporters inside it, removing the requirements for Raman reporters to have anchoring groups or charges.	173
